# New Treatment for Tape Worm

**Published:** 1869-02

**Authors:** 


NEW TREATMENT FOR TAPEWORM.—Dr. Lurtel (*Gazette Medicale de Paris*) has tried with success the following method: He gives in one dose two-thirds of an ounce of ether, followed two hours afterwards by an ounce of castor oil. The worm is discharged entire or almost so, and always with the head intact. No pain is caused by this treatment.

